# Settings, populations, and time: a conceptual framework for public health interventions

**DOI:** 10.3389/fpubh.2023.1297019

**Published:** 2023-12-15

**Authors:** Jens Aagaard-Hansen, Paul Bloch

**Affiliations:** ^1^Diabetes Prevention Research, Department for Prevention, Health Promotion and Society, Steno Diabetes Center Copenhagen, Herlev, Denmark; ^2^SA MRC Developmental Pathways for Health Research Unit, Faculty of Health Sciences, University of the Witwatersrand, Johannesburg, South Africa

**Keywords:** health promotion, life-course perspective, planning, population segments, public health, setting approach

## Abstract

This paper presents a conceptual framework displaying how combinations of settings and populations seen in a long-term perspective may guide public health and health promotion planning and research. The notion of settings constitutes a key element of health promotion as stipulated by the Ottawa Charter from 1986. The setting approach highlights the individual, social and structural dimensions of health promotion. Likewise, the notion of populations and how they are selected forms a center pillar of public health. By joining the two perspectives, four combinations of intervention strategies appear by addressing: (1) a single population segment within a single setting, (2) multiple population segments within a single setting, (3) a single population segment within multiple settings or (4) multiple population segments within multiple settings. Furthermore, the addition of a time dimension inspired by the life-course perspective illustrates how trajectories of individuals and projects change settings and population segments as time goes by. The conceptual framework displays how systematic awareness of long-term, multi-setting, multi-population trajectories allow health promotion planners and researchers to systematically develop, plan and analyze their projects.

## Introduction

This paper introduces a conceptual framework showing how combinations of single or multiple settings and population segments may be selected to guide long-term strategic public health and health promotion policy, planning and research.

### The setting perspective

The Ottawa Charter remains the key value foundation for working with health promotion (1). It defines health promotion as “the process of enabling people to increase control over, and to improve, their health” (1), p. 1. Moreover, it conceptualizes health promotion action as building healthy public policy; creating supportive environments; strengthening community actions; developing personal skills; reorienting health services (beyond its responsibility for providing clinical and curative services) and moving into the future (with caring, holism and ecology as central strategic elements) (1).

The Ottawa Charter further specifies that health promotion action “has to be facilitated in schools, homes, workplaces and community settings” because “health is created and lived by people within the settings of their everyday life; where they learn, work, play and love” (1), p. 4. WHO defines a setting as a “place or social context in which people engage in daily activities in which environmental, organizational and personal factors interact to affect health and well-being” (2), p. 30. A setting is also where people actively use and shape the environment and thus create or solve problems relating to health. Poland and colleagues further argue that settings are both the medium and the product of human social interaction and, thus, more than simply locations in space–time (3). Kokko and Baybutt (4) provide an overview of more recent academic publications on the theoretical basis and practical principles of setting-based health promotion. Consequently, the setting approach emphasizes the individual, social and structural dimensions of health promotion (5, 6).

### Populations

The notion of population is key to public health interventions and may be determined according to biomedical, social, spatial, or temporal criteria (7). Geoffrey Rose (8) juxtaposed two population-related concepts. “On the one hand many programs focus a certain risk factor (e.g., smoking) or biomarker (e.g., elevated blood pressure identified through screening) thereby identifying a segment of the total population” (7), p. 2. On the other hand, the alternative ‘whole population approach’ describes interventions that in principle impact everyone (e.g., fiscal policies of tobacco taxation). “According to Rose the overall sum of the impact on the “whole population” is larger than the total impact on the group of “individuals at risk” “(7), p. 2 – which is likely to provide higher return of investment.

Based on Phelan and Link’s focus on ‘fundamental causes’ (9), Frohlich and Potvin (10) criticized this approach. They noted “that a whole population approach does not address the underlying determinants, and that it is likely to increase health inequalities due to uneven distribution of risk factors as well as in disparate ability to benefit from interventions” (7), p. 2. Instead Frohlich and Potvin recommended a focus on ‘vulnerable populations’, defined as a ‘subgroup or subpopulation who, because of shared social characteristics, is at higher risk of risks’ (10), p. 218.

Although interventions may target individuals or population segments within given settings, more structured public health measures are also needed to ensure healthy lives for all (11). Thus, some health promotion endeavors such as health promoting changes in fiscal policy (e.g., increased taxation of tobacco or sweet beverages) work on whole populations irrespectively of settings and life stages. The ongoing discussion on proportionate universalism explores how such interventions should be designed with a view to reduce (and not increase) health inequity (12, 13).

### The life-course perspective

In recent years the concept of Developmental Origins of Health and Disease (DOHaD) has illustrated the importance of the life-course perspective derived from combined evidence from long-term epidemiological studies and epigenetic research (14, 15). The concept describes how positive and negative factors throughout an individual’s life-course (but especially during ‘the first 1,000 days’ including the gestational period) determine risk accumulation of many diseases as well as socioeconomic and educational outcomes (16, 17). For example, harmful events occurring early in the life-course can lead to reduced cognitive ability, educational outcomes, and lifetime earnings (18) as well as an increased risk of non-communicable diseases (19). In addition to this biomedical life-course perspective, a substantial amount of related research has been conducted within social sciences, which will not be elaborated here (20, 21).

## Four combinations of settings and populations in intervention strategies

**Figure 1:**
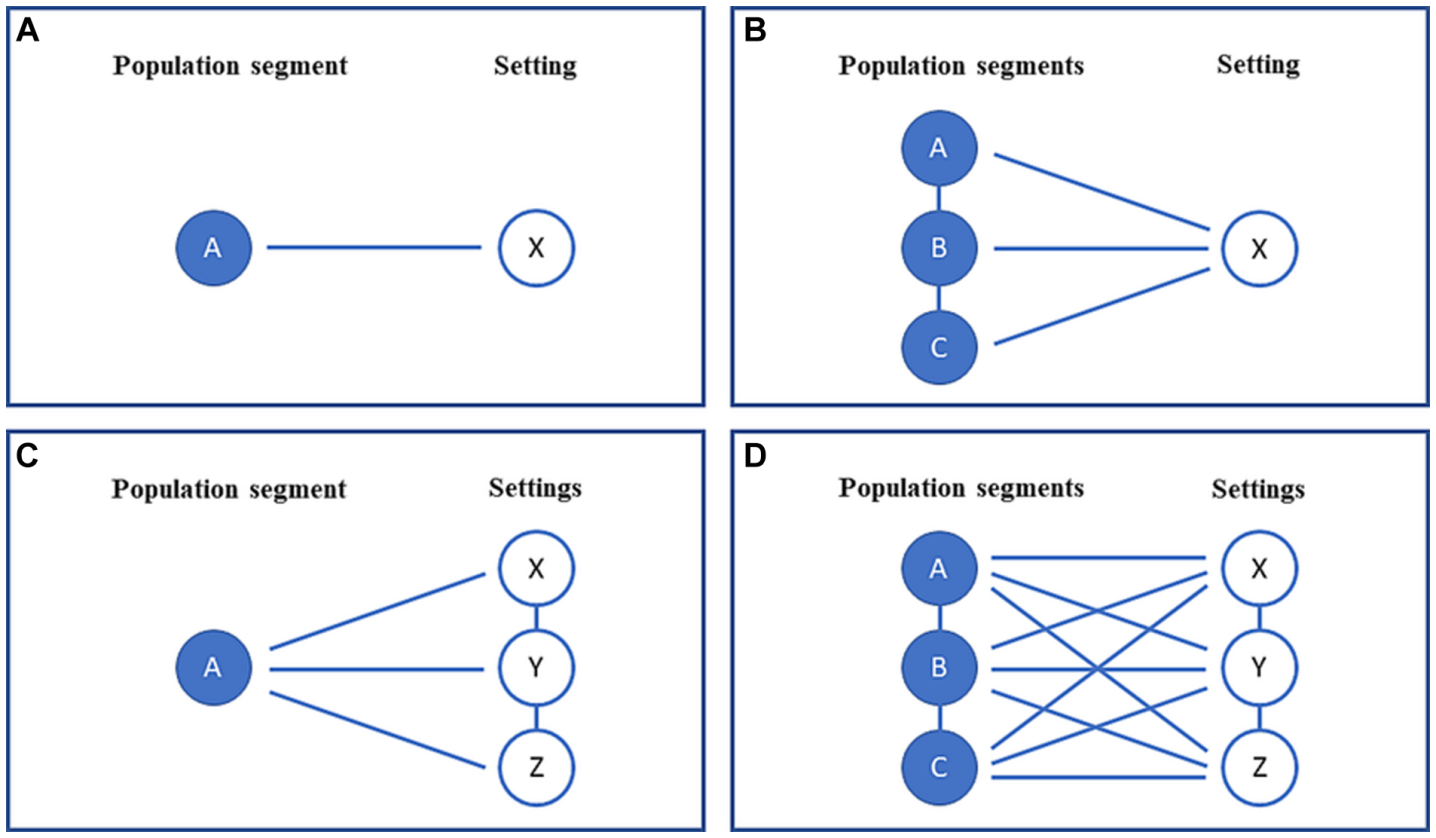
**(A–D)** A conceptual framework illustrating the four possible combinations of single or multiple populations and settings applicable to public health interventions.

Theoretically, the notions of settings and populations can be combined in four different intervention strategies as illustrated in Figure 1. Thus, four possibilities appear: (1) a single population segment within a single setting, (2) multiple population segments within a single setting, (3) a single population segment within multiple settings or (4) multiple population segments within multiple settings. By a population segment we mean a sub-population selected based on either demographic, social or other criteria.

### A single population segment within a single setting

This constitutes the simplest form of intervention comprising one population segment and one setting (Figure 1A), as exemplified by Case 1.

Case 1 **Workplace health promotion in Japan (22).**

The intervention took place in 2005 at a large Japanese manufacturing enterprise comprising several worksites, engineering, and clerical departments. The aim was to improve the working environment and the workers’ mental health (22). The intervention was conducted by a work improvement team and applied a self-reported questionnaire with a list of 30 items after which planning workshops were being held. There were several significant positive effects for female employees assessed by the Brief Job Stress Questionnaire, including skill underutilization, supervisor and co-worker support, psychological distress, and job satisfaction, whereas there were no equivalent improvements for male workers. This project is an example of an intervention within one setting (a company) addressing one population group (the workers) here defined according to a social criterion (22).

### Multiple population segments within a single setting

Figure 1B displays how an intervention may involve more than one population segment within a single setting. This is illustrated by Case 2.

Case 2 **The whole school approach (23).**

The notion of ‘health promoting schools’ recommends a focus on six components: (1) healthy school policies; (2) school physical environment; (3) school social environment; (4) individual health skills and action competence; (5) community links and (6) health services (23). Evidently, school aged children are in focus, but in the comprehensive interpretation even teachers and parents are within the scope of the interventions, making it a multiple population segment - one setting endeavor. This is an example of a health promotion approach operating within one setting (a school) addressing more than one group (students, teachers, and parents) (23).

### A single population segment within multiple settings

Figure 1C displays how a single group may be reached by an intervention within more than one setting, as shown in Case 3.

Case 3 **Project SoL: Health and Local Community (24).**

Project SoL was a complex community-driven health promotion intervention that aimed to promote healthy living among Danish families with young children (24). ‘SoL’ is an abbreviation of ‘Sundhed og Lokalsamfund’ in Danish or ‘Health and Local Community’ in English. The focus of the project was to promote healthier dietary habits and physical activity, as well as to mobilize local community resources, strengthen social networks and reduce social inequity. Project SoL was carried out from 2012 to 2015 within multiple settings including schools, day care centers, supermarkets, and local mass media, but also within local community settings more widely in three small towns on the Danish island of Bornholm (24). The intervention was co-created together with professional stakeholders and citizens using action research methodology and was implemented in a coordinated and integrated manner in several everyday life settings to promote intensity, synergy, and sustainable impact (24). Thus, the SoL project involved one population group (families with young children) within multiple settings in the local community.

### Multiple population segments within multiple settings

Figure 1D illustrates the most complicated situation where a project combines multiple population segments and settings, as illustrated by Tingbjerg Changing Diabetes initiative in Case 4.

Case 4 **Tingbjerg Changing Diabetes (25, 26).**

Tingbjerg Changing Diabetes (TCD) is a long-term community-based initiative that applies the supersetting approach (26) to promote health and prevent type 2 diabetes among high-risk population groups living in the disadvantaged neighborhood of Tingbjerg in Copenhagen, Denmark (25). “TCD constitutes a strategic, organizational, and locational framework for developing, implementing, evaluating, and improving a variety of interventions, projects and activities in a local community together with citizens, professional practitioners, researchers and decision-makers in public institutions, private enterprises and civic organizations” (26), pp. 2–3.

Following the principles of the supersetting approach, “TCD includes multiple coordinated interventions driven by multiple intersectoral stakeholders and participants in multiple local community settings. TCD addresses the social challenges of people’s everyday lives in efforts to empower them to act for better health and well-being for themselves, their families, and their community” (27). In this way, it is fundamental to the intervention to both identify and mobilize community assets and resources to support health and well-being among people with the aim to increase their control over their health and their community (27). Thus, TCD is an example of a multi-population, multi-setting approach.

## Adding the time perspective

The typology shown in Figure 1 outlines four theoretical combinations of settings and populations. However, as they stand, they are static. The addition of a long-term longitudinal angle inspired by the life-course perspective adds new insights. The life-course perspective provides a chronological dimension to our conceptual framework by spanning fetal life, infancy, early childhood, school age, adolescence, reproductive age, and old age (28). Whereas the original focus of the DOHaD discourse has been on the accumulation of risk and protective factors for various conditions over the life-course and across generations, its main contribution in this article is to remind us of the importance of applying a long-term perspective from cradle to grave.

### Changing settings in a life-course perspective

**Figure 2:**
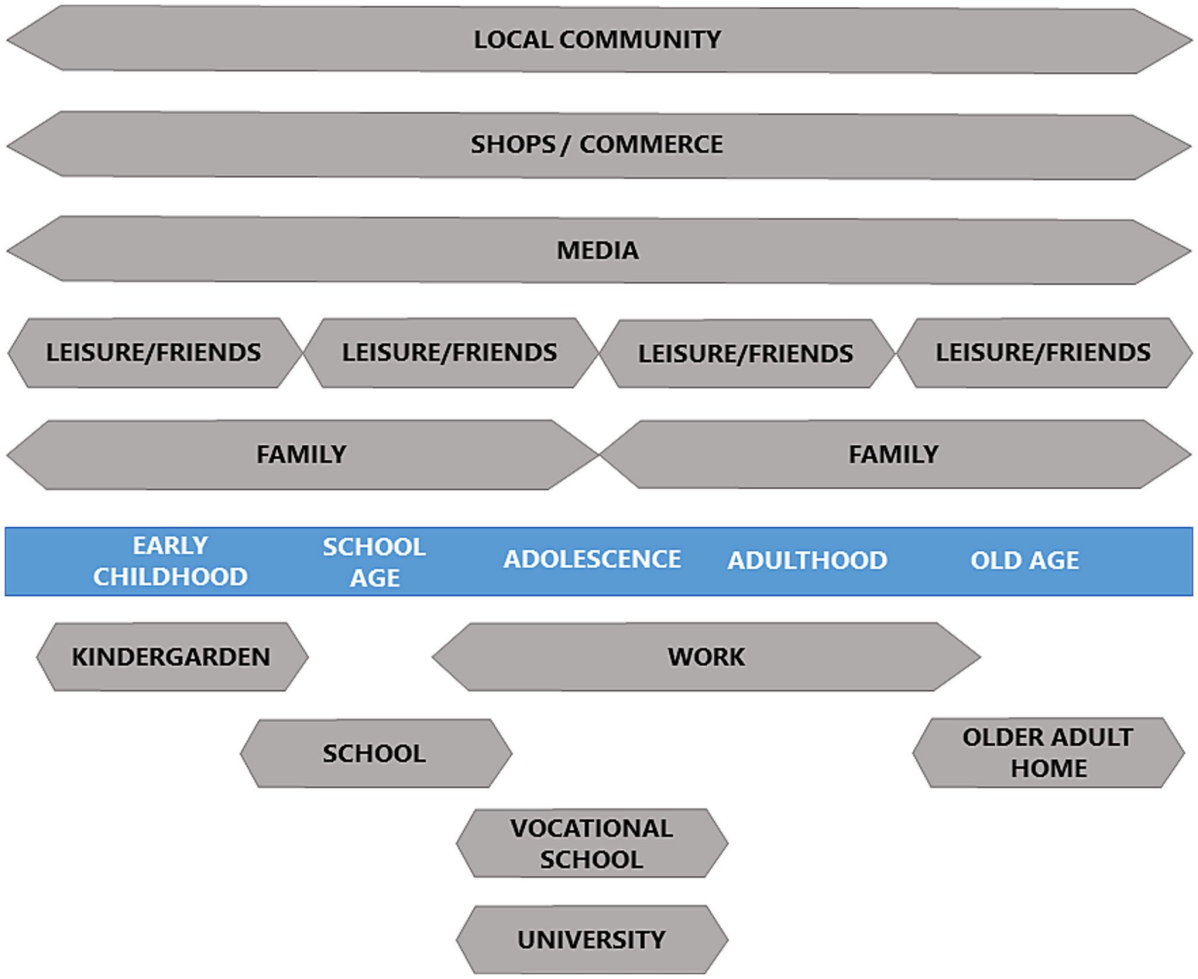
A prototype showing relevant settings across the life-course in a Northern European context.

Figure 2 illustrates how settings change according to the stages of an individual’s life-course (indicated by the blue band). Hence, for each population segment (age group at a certain stage of the life-course) there is a certain set of relevant settings. For instance, in school age it is shown how the school, family and leisure/friends play key roles. A bit later in life the learning institutions have changed to either vocational schools or universities and workplaces come into the picture, whereas the family as well as leisure/friends still plays a role. As indicated by the graphics, there may be changes in various settings. Thus, for instance the group of friends may also change according to life stages, as they may be derived from the other settings such as schools, workplaces, or leisure activities. During time, local community, shops/commerce, and media serve as important settings though their contexts may change as well.

Throughout life, the family usually plays a special role though they also change over time. When individuals reach adulthood, they usually establish their own families with spouses and children in addition to the parents and siblings. A more simplified version of this idea was suggested in 2010 (29), p. 42.

The notion of setting reminds us that individuals and populations live in contexts of built and social environments that have positive or negative impacts on their health, and which must consequently be taken into consideration when planning public health programs (26, 30).

## A dynamic perspective on public health interventions

The original focus of the life-course perspective is to highlight the accumulation of risk during life trajectories thereby differentiating various age (population) groups (7). But it also serves as a reminder of change over time, which applies in other ways.

A given setting may, thus, undergo changes over time. For instance, within urban environments, a given neighborhood may slowly change from prestigious high-end living spaces to deprived areas or vice versa depending on several political, demographic, and socio-economic factors. In addition, the populations living within settings will change as well due to demographic trends including migration.

Moreover, a project may have its own ‘life-course’ irrespective of whether its focus is on public health practice or research. Thus, an intervention may start out dealing with one population segment within one setting (Figure 1A) and gradually expand to one of the more complex types illustrated (Figures 1B–D).

To our knowledge, this is the first time that a conceptual framework systematically explores the theoretical links between the (multi-)setting and (multi-)population approaches seen in a life-course perspective.

Especially the settings diagram is highly context specific depending on regional, national and even local variations based on socio-economic, ethnic, religious and other social determinants. Thus, the graphics displayed in Figure 2 may be claimed to be relevant for a mainstream, Northern European context. Nevertheless, the principles are of a general nature and can easily be adapted globally to other locations and circumstances.

We recommend that future research should aim to explore these large-scale and long-term spatial and temporal trajectories and patterns of change which are likely to evade more limited short-term projects. This will require willingness of research sponsors to support such comprehensive endeavors and a change of the mindset of some research groups as well.

## Conclusion

This paper has introduced a conceptual framework combining the notions of settings and population segments seen in a longitudinal perspective inspired by the life-course perspective to guide public health and health promotion policy, planning and research. This perspective provides a typology that enables planners and researchers to obtain a comprehensive picture of populations in spatial (setting) and temporal (life-course) dimensions of public health and health promotion.

## Data availability statement

The original contributions presented in the study are included in the article/supplementary material, further inquiries can be directed to the corresponding author.

## Author contributions

JA-H: Conceptualization, Writing – original draft, Writing – review & editing. PB: Conceptualization, Writing – original draft, Writing – review & editing.
